# 398. Influence of environmental factors over the incidence of histoplasmosis: A Pilot Study in 3 Midwestern States

**DOI:** 10.1093/ofid/ofaf695.136

**Published:** 2026-01-11

**Authors:** Julio C Zuniga-Moya, Benjamin Papadopoulos, Jennifer R Head, Adriana Rauseo, Patrick B Mazi, Leda Kobziar, Andrej Spec, Andrew Atkinson

**Affiliations:** Washington University School of Medicine in St. Louis, St. Louis, Missouri; Washington University in St Louis School of Medicine, St Louis, Missouri; University of Michigan, Ann Arbor, Michigan; Washington University in St. Louis, Saint Louis, MO; Washington University, St Louis, Missouri; University of Idaho, Coeur d'Alene, Idaho; Washington University School of Medicine in St. Louis, St. Louis, Missouri; Washington University School of Medicine, St. Louis, Missouri

## Abstract

**Background:**

Histoplasmosis is a potentially fatal fungal infection if not diagnosed promptly and treated. The literature remains unclear regarding which environmental conditions favor its transmission, growth, and incidence.

**Figure:**
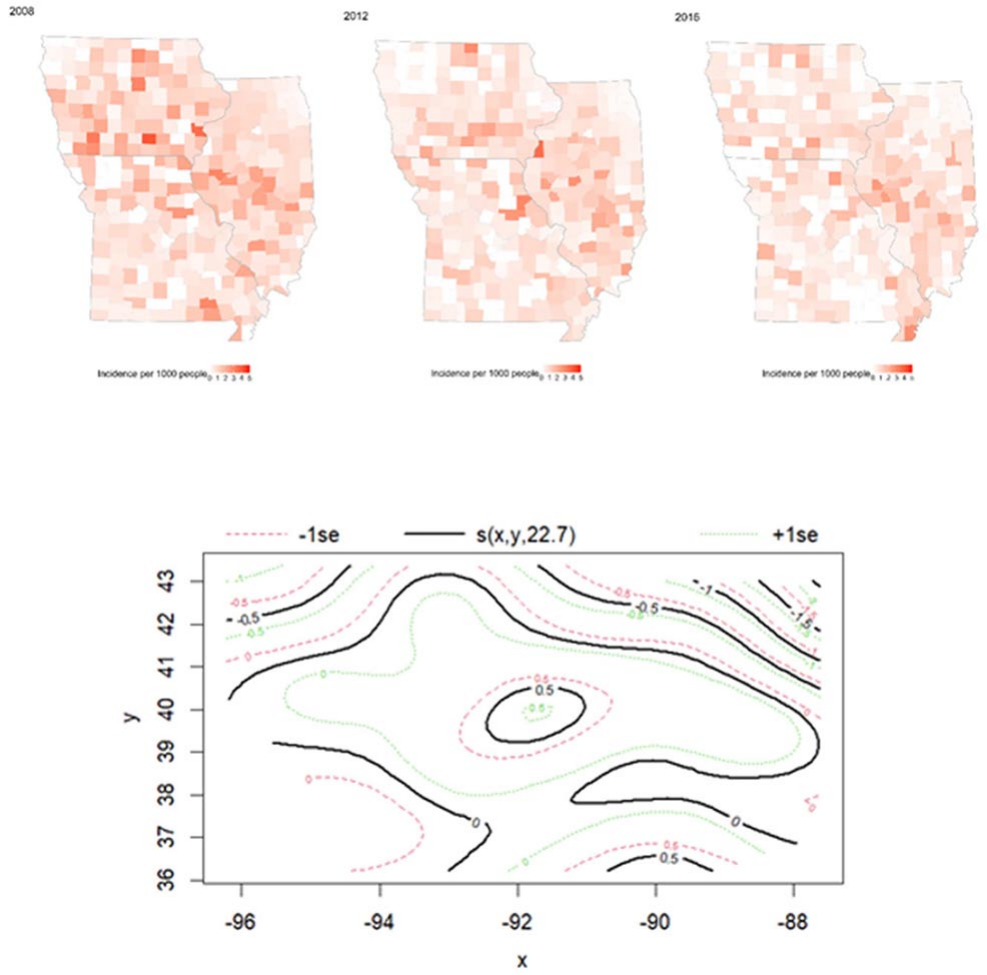
Histoplasmosis incidence 2008-16 in Illinois, Iowa and Missouri. Top: Crude annual incidence per 1000 people, by county; Bottom: Contour map of estimated incidence from fitted GAM model for the spline smoother of longitude (x) and latitude (y) (all years); s(x,y, 22.7) denotes incidence estimate relative to the average (with degrees of freedom used (black)) along with confidence band of +1 standard error (SE, green) and -1 SE (red); central region has highest incidence, along with one in the extreme bottom right corner.

**Methods:**

The goal of the study was to investigate the association between the incidence of histoplasmosis and temperature, rainfall and drought. In this pilot study, we utilized the Medicare Fee-for-Service database to access information on diagnoses of histoplasmosis between 2008 and 2016 among patients aged 65 and older living in Iowa, Illinois, and Missouri. We aggregated cases by county of patient residence and month of diagnosis. We fitted mixed effects Poisson general additive spatial models with the dependent variable the number of cases per county per month. Models included log of population as offset term, and splines on the geographical coordinates for the centroid of the county, along with year (and their interaction), with a random intercept term for each county. We included fixed effects for season, state, proximity to the Mississippi River, presence of drought conditions and included splines for mean monthly temperature and precipitation.

**Results:**

There were 12,495 diagnoses of histoplasmosis in 316 counties (102 IL, 99 IA, 115 MO) over 9 years, with considerable variability between counties (median incidence 0.45 cases per 1000 people, interquartile range (IQR) [0.19, 0.70], Figure 1). Greater distance from the river (“two or more counties removed” compared to “on the river”, adjusted incidence rate ratio (aIRR): 1.13; 95% CI: 1.03-1.24], p=0.008) and higher mean temperature increased risk (+1°C aIRR 1.02 [1.01, 1.04], p=0.01), whereas drought (aIRR 0.90, [0.83, 0.97], p=0.008) and being located in Missouri (aIRR 0.64 [0.51, 0.81], p< 0.001) reduced risk.

**Conclusion:**

Our findings suggest that higher average monthly temperature is associated with higher incident cases of histoplasmosis in the studied area, but drought conditions and proximity to river are also important. Based on our findings, histoplasmosis risk is multifactorial, and future research will investigate the complex interactions in more detail, with a view to identify and develop strategies to reduce or manage the incidence of histoplasmosis.

**Disclosures:**

All Authors: No reported disclosures

